# Associations between Nine Polymorphisms in EXO1 and Cancer Susceptibility: *A Systematic Review and Meta-Analysis of 39 Case-control Studies*

**DOI:** 10.1038/srep29270

**Published:** 2016-07-08

**Authors:** Meng Zhang, Duran Zhao, Cunye Yan, Li Zhang, Chaozhao Liang

**Affiliations:** 1 Department of Urology, the First Affiliated Hospital of Anhui Medical University, Hefei, China; 2 Institute of Urology, Anhui Medical University, Hefei, China

## Abstract

An increasing number of studies have highlighted the potential link between EXO1 polymorphisms and cancer risk, although no consensus has yet been obtained. Thus, we aimed to obtain a thorough and current assessment of EXO1 polymorphisms and cancer susceptibility by performing a meta-analysis. A comprehensive literature retrieval was performed on PubMed, EMbase, Web of Science and Wanfang databases. The odds ratio (OR) and 95% confidence interval (CI) were applied to assess the results. Finally, 39 case-control studies of the nine EXO1 polymorphisms that involved 21,651 cases and 21,348 controls met our inclusion criteria. The pooled analysis indicated that the rs1047840 polymorphism conferred a significantly increased susceptibility to cancer in an allelic model. Similarly, the rs3754093, rs1776177, rs9350, rs10802996, rs1635498, rs1776148 and rs851797 polymorphisms were also associated with an increased susceptibility to cancer in an allelic model, respectively, while no significant association was identified for rs1635517 polymorphism. For the rs1047840 polymorphism, in an ethnicity subgroup analysis, a significantly increased susceptibility to cancer for Asians was identified in all the genetic models, and for Caucasians in an allelic model. Our findings provide the evidence that the rs1047840, rs9350, rs10802996, rs1635498, rs1776148, rs1776177, rs3754093 and rs851797 polymorphisms may act as risk factors for cancer.

Currently, cancer is a primary cause of human death, which can be attributed to its high rates of morbidity and mortality in the United States and many other countries^1^. Large epidemiological and clinical investigations had indicated that a multitude of factors contribute to the initiation of tumourigenesis, such as environmental factors, hereditary factors and cancer-related lifestyle factors. Additionally, susceptibility genes, including EXO1^2^, have been found to play a key role in the initiation of cancer.

The Exonuclease 1 (EXO1) gene, which belongs to the RAD2 nuclease family, encodes a member of the mismatch repair (MMR) system that plays a critical role in maintaining genomic stability^3^. EXO1 is located on chromosome 1q42–q43, includes one untranslated exon and 13 coding exons, and encodes an 846 amino acid protein. The products of the EXO1 gene function in DNA replication, repair, mutation avoidance and recombination, which are necessary processes for both male and female meiosis^4^.

Recently, the associations between EXO1 genetic polymorphisms and susceptibility to various type of cancers had been widely investigated. An EXO1 polymorphism at codon 589 (rs1047840) is a non-synonymous single nucleotide polymorphism (SNP) that has been associated with susceptibility to lung cancer (LC)^5^,^6^,^7^, glioma^8^, breast cancer (BC)^9^, and gastric cancer (GC)^10^. As such, it may be a novel useful marker for primary tumour prevention and anticancer interventions. However, other common low-penetrance susceptibility alleles may also exist, which lead to a moderate increase or reduction in cancer susceptibility. To date, only a few molecular epidemiological studies have investigated other EXO1 polymorphisms and cancer susceptibility in various populations, such as A-1419G (rs3754093), G670E (rs1776148), C498T (rs1635517), and L757P (rs9350). Additionally, no consensus had yet been obtained, which was partially a consequence of the heterogeneity within cancer subtypes, the diverse ethnicity of patient cohorts, and the small sample sizes. In the present meta-analysis, we had widely reviewed all eligible publications that were based on case-control data to derive a more precise and up-to-date estimation of associations between polymorphisms in EXO1 and cancer susceptibility.

## Methods

### Literature search and eligibility

We performed a comprehensive literature search using the PubMed, Web of Science, EMbase and Wangfang databases (last research update: September 29, 2015) in which we applied the following search terms: (EXO1 OR exonuclease 1) AND (polymorphism OR SNP OR variant OR mutation OR allele) AND (cancer OR tumour OR carcinoma OR neoplasm OR malignancy). We also manually retrieved reference lists from these enrolled publications, aiming to ensure that all eligible studies were included.

### Inclusion and exclusion criteria

The detailed inclusion criteria were as follows: 1) the study was a case-control study; 2) the study evaluated the association between EXO1 polymorphisms and cancer susceptibility; 3) the study comprised useful allele and genotype frequencies to estimate the crude ORs at 95% CIs. However, all meta-analyses, reviews, animal studies and case-only studies, as well as those duplicated previous publications, were definitely excluded. Studies deviated from Hardy Weinberg Equilibrium (HWE), studies that were not concerned with cancer susceptibility and abstracts with incomplete genetic data were also removed from this analysis. When a case group was investigated in more than one publication, the publication with the largest number of participants was selected.

### Data extraction

The following information was extracted from each study by two independent investigators (Meng Zhang and Duran Zhao): name of the first author, year of publication, ethnicity, genotyping methods, source of controls, cancer type, total number of cases and controls, and HWE. Any discrepancies between the two investigators’ selections were resolved by consensus. Regarding the sources of controls, all eligible case-control studies were defined as either population-based (PB) or hospital-based (HB).

### Statistical analysis

Susceptibility to cancer related to EXO1 polymorphisms was calculated directly from data provided in the eligible studies. Crude OR corresponding to 95% CI was applied to evaluate the strength of association between EXO1 polymorphisms and cancer susceptibility. Variants in EXO1 included rs1635498, rs1047840, rs851797, rs3754093, rs1776177, rs1776148, rs1635517, rs10802996 and rs9350. Each variable was analysed in allelic comparison, heterozygote comparison, homozygote comparison, dominant and recessive models. For example, the pooled ORs of EXO1 rs9350 polymorphisms were calculated by allelic comparison (T vs. C), heterozygote comparison (TC vs. CC), homozygote comparison (TT vs. CC), dominant (TT + TC vs. CC) and recessive models (TT vs. TC + CC). We used a χ^2^-based Q-test to evaluate between-study heterogeneity within the studies^11^. Heterogeneity was considered to be significant when the *P*-value was less than 0.1. If there was no significant heterogeneity, a fixed effect model (Der-Simonian Laird) was used to evaluate the point estimates and 95% CI; otherwise, a random effects model (Der-Simonian Laird) was used^12^. The *Z*-test was used to determine the significance of overall ORs. In addition, Bonferroni corrections were also performed to adjust the results^13^. If significant heterogeneity existed among the enrolled case-control studies, meta-regression was used to elaborate the source of heterogeneity with the Stata version 12.0 software. Moreover, one-way sensitivity analyses were carried out to evaluate the stability of the pooled ORs, in which each individual study was removed from the meta-analysis to detect the effect of each individual data set on the pooled ORs. Publication bias was evaluated using Begg’s funnel plot and Egger’s test based on Stata 12.0 software^14^.

### Linkage disequilibrium (LD) analysis across populations

We extracted data from the 1000 genomes Project Phase III (http://hapmap.ncbi.nlm.nih.gov/cgi-perl/gbrowse/hapmap3r2_B36/) encompassing the polymorphisms in EXO1 evaluated in currently study^15^. Briefly, populations enrolled in the project including ASW (African ancestry in Southwest USA), CEU (Utah residents with Northern and Western European ancestry from the CEPH collection), CHB (Han Chinese in Beijing, China), CHD (Chinese in Metropolitan Denver, Colorado), GIH (Gujarati Indians in Houston, Texas), JPT (Japanese in Tokyo, Japan), LWK (Luhya in Webuye, Kenya), MEX (Mexican ancestry in Los Angeles, California), MKK (Maasai in Kinyawa, Kenya), TSI (Toscans in Italy) and YRI (Yoruba in Ibadan, Nigeria). Then, Haploview software was used to perform analysis and LD was evaluated by r^2^ statistics^16^ in each of the above mentioned populations.

## Results

### Study characteristics

**Figure 1:**
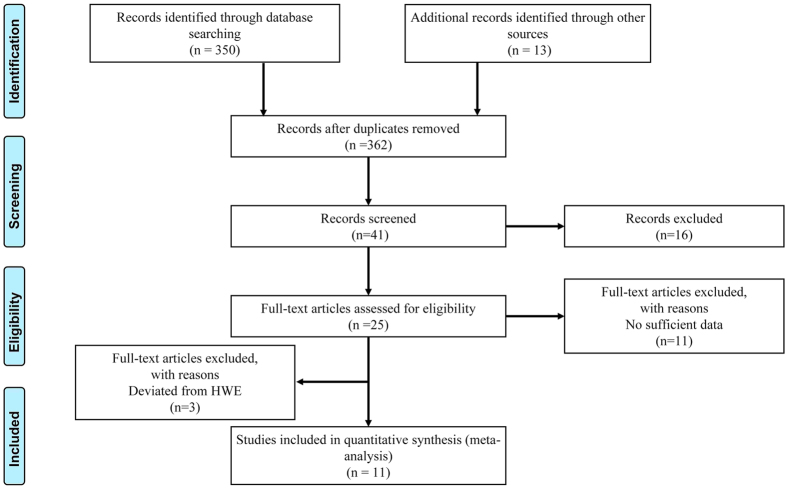
Flow chart of studies selection in this meta-analysis.

As shown in Fig. 1, a total of 362 citations were retrieved from database searches. After reading the title or abstract, 41 studies concerning the associations of the nine EXO1 polymorphisms and cancer susceptibility were selected for further consideration. Then, 27 articles were removed because they were duplicated studies, lacked sufficient genotype information, or used ineligible samples. Finally, we collected 14 publications that encompassed 50 case-control studies, including 28,462 cases and 28,253 controls of the nine polymorphisms in EXO1 (rs1635498, rs1047840, rs851797, rs3754093, rs1776177, rs1776148, rs1635517, rs10802996 and rs9350) (Table 1)^4^,^5^,^6^,^8^,^9^,^10^,^17^,^18^,^19^,^20^,^21^,^22^,^23^,^24^. The quality of these enrolled case-control studies was evaluated using the Newcastle–Ottawa Scale (NOS) (Supplementary Table 1). Genotypic distribution of most of the studies was in agreement with HWE (*P* > 0.05) in controls population except for 11 case-control studies^5^,^6^,^9^,^10^,^17^,^19^,^23^, which were eventually excluded from the pooled analyses. In the end, a total of 11 publications, encompassing 39 case-control studies of the nine polymorphisms that involved 21,651 cases and 21,348 controls were finally enrolled.

For the rs1047840 polymorphism, 11 case-control studies with 6,289 cases and 6,333 controls met our inclusion criteria. Among these, four studies were of Caucasian individuals, five were of Asians and others were of mixed groups. For the rs9350 polymorphism, five case-control studies, which included 3,173 cases and 2,895 controls were enrolled. Among these, only one study was performed in a Caucasian cohort, while the others considered as Asian individuals. For rs1776177 polymorphism, three case-controls of Asians that encompassed 1,217 cases and 1,217 controls were enrolled. For the rs1776148 polymorphism, we ultimately enrolled two case-control studies of Caucasians that included 711 cases and 490 controls. For the rs10802996 polymorphism, two case-control studies of Asians that included 305 cases and 457 controls met our eligibility criteria. Moreover, for the rs851797, rs3754093, rs1635517 and rs1635498 polymorphisms, there were four case-control studies with 2,489 cases and 2,489 controls that were included in this present meta-analysis, and the ethnicity of these studies were of Asian populations.

### Quantitative synthesis

**Figure 2:**
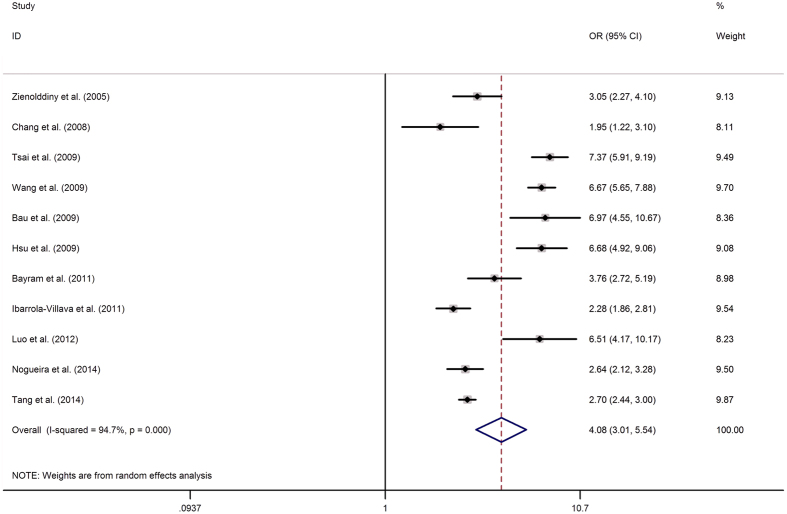
Forest plots of the association between EXO1 rs1047840 polymorphism and cancer susceptibility (allelic comparison A vs. G). Each square indicate a study, and the area of squares is proportional to the weight of the study. The diamond represents the summary OR and 95% CI. CI = confidence interval, OR = odds ratio.

**Figure 3:**
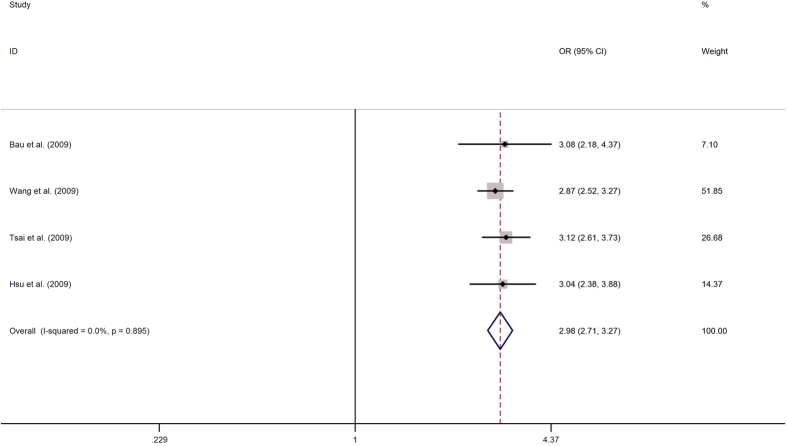
Forest plots of the association between EXO1 rs3754093 polymorphism and cancer susceptibility (allelic comparison G vs. A). Each square indicate a study, and the area of squares is proportional to the weight of the study. The diamond represents the summary OR and 95% CI. CI = confidence interval, OR = odds ratio.

The results of rs1047840 and rs9350 polymorphisms and cancer risk were shown in Table 2, and the results of other polymorphisms were shown in Supplementary Table 2. Overall, the pooled analysis indicated that the A allele (variant allele) of the rs1047840 polymorphism conferred a significantly increased overall susceptibility to cancer in an allelic model (A vs. G: OR = 4.082, 95% CI = 3.009–5.538, *P* = 0.000, Fig. 2). Similarly, rs3754093 and rs1776177 polymorphisms were related to an increased susceptibility to cancer in an allelic model (rs3754093: G vs. A: OR = 2.976, 95% CI = 2.711–3.268, *P* = 0.000, Fig. 3; rs1776177: G vs. A: OR = 3.234, 95% CI = 2.815–3.716, *P* = 0.000). Additionally, rs9350, rs10802996, rs1635498, rs1776148 and rs851797 polymorphisms were also conferred an increased overall susceptibility to cancer in an allelic model (rs9350: T vs. C: OR = 2.930, 95% CI = 2.124–4.042, *P* = 0.000; rs10802996: G vs. C: OR = 5.013, 95% CI: 3.717–6.762, *P* = 0.000; rs1635498: G vs. A: OR = 7.965, 95% CI: 6.924–9.163, *P* = 0.000; rs1776148: G vs. A: OR = 1.448, 95% CI: 1.209–1.734, *P* = 0.000; rs851797: T vs. C: OR = 1.841, 95% CI: 1.686–2.009, *P* = 0.000). However, no significant association was identified for rs1635517 polymorphism and overall cancer susceptibility.

### Subgroup analyses

Results of subgroup analyses were presented in Table 2. In an ethnicity subgroup analysis, a significantly increased susceptibility to cancer in Asian populations was identified in all the genetic models for rs1047840 polymorphism, while an increased susceptibility to cancer was identified for Caucasian populations only in an allelic model (A vs. G: OR = 2.709, 95% CI = 2.075–3.538, *P* = 0.000). When the subgroup analysis was conducted by source of control, an increased susceptibility to cancer was identified for P-B groups in an allelic model (A vs. G: OR = 4.357, 95% CI = 3.172–5.986, *P* = 0.000). In addition, we uncovered an increased susceptibility to lung cancer (LC) in an allelic model (A vs. G: OR = 4.510, 95% CI = 2.094–9.713, *P* = 0.000). In the stratification analysis by genotyping methods, an increased susceptibility to cancer was revealed for PCR-RFLP (A vs. G: OR = 5.327, 95% CI = 3.631–7.815, *P* = 0.000; AA + AG vs. GG: OR = 1.405, 95% CI = 1.144–1.726, *P* = 0.001) and SNP-Chip (A vs. G: OR = 2.488, 95% CI = 1.881–3.292, *P* = 0.000) groups, respectively.

For rs9350 polymorphism, when the stratification analysis was conducted based on genotyping method, we also identified a significant increased susceptibility to cancer in PCR-RFLP group in an allelic model (T vs. C: OR = 2.412, 95% CI = 2.201–2.643, *P* = 0.000) for Asian population.

### Sensitivity analyses and publication bias

**Figure 4:**
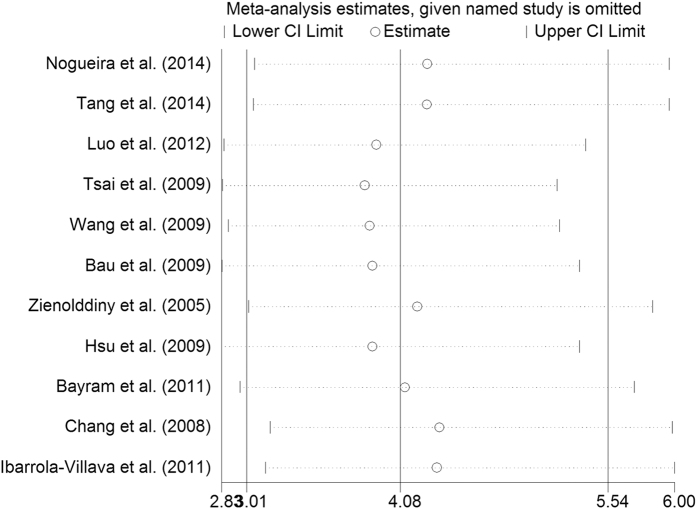
Sensitivity analysis of EXO1 rs1047840 polymorphism and overall cancer susceptibility (allelic comparison A vs. G).

**Figure 5:**
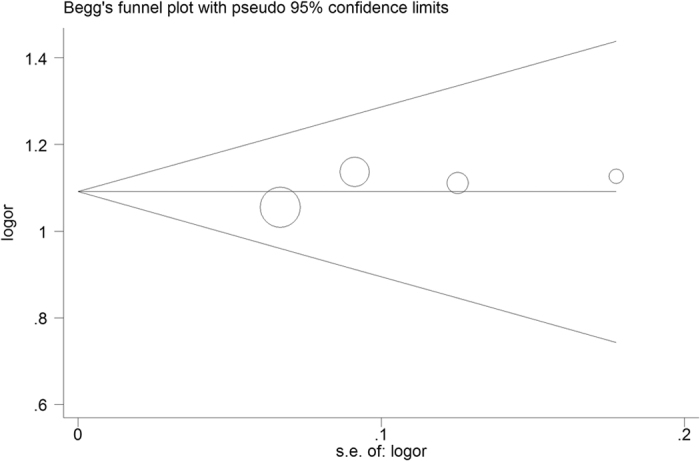
Begg’s funnel plot for publication bias test under EXO1 rs3754093 (allelic comparison G vs. A). The x-axis is log (OR), and the y-axis is natural logarithm of OR. The horizontal line in the figure represents the overall estimated log (OR). The two diagonal lines indicate the pseudo 95% confidence limits of the effect estimate. Log (OR) = log-transformed OR, OR = odds ratio.

**Figure 6:**
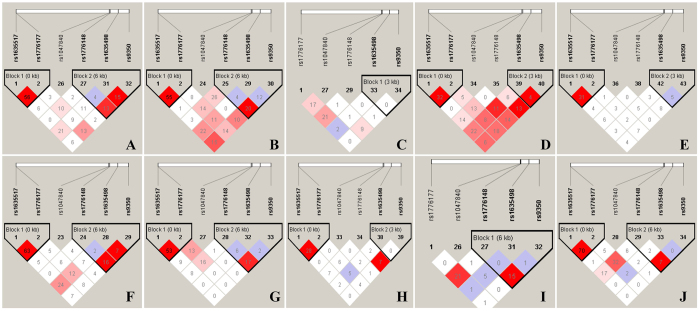
Linkage disequilibrium analyses for EXO1 polymorphisms in populations from 1000 genomes Phase 3. The number of each cell represents r^2^ and white color cells shows no LD between polymorphisms. Population descriptors: ASW: African ancestry in Southwest USA, CEU: Utah residents with Northern and Western European ancestry from the CEPH collection, CHB: Han Chinese in Beijing, China, CHD: Chinese in Metropolitan Denver, Colorado, GIH: Gujarati Indians in Houston, Texas, JPT: Japanese in Tokyo, Japan, LWK: Luhya in Webuye, Kenya, MEX: Mexican ancestry in Los Angeles, California, MKK: Maasai in Kinyawa, Kenya, TSI: Toscans in Italy, YRI: Yoruba in Ibadan, Nigeria. The rs numbers are SNP IDs taken from National Center for Biotechnology Information (NCBI).

Sensitivity analysis was performed by excluding each single case-control study in turn, and no individual study showed a significant influence on the pooled ORs. Sensitivity analysis of the rs1047840 polymorphism in an allelic comparison is presented in Fig. 4 (Supplementary Table 3). Additionally, Begg’s funnel plot was generated and Egger’s test was performed to assess potential publication bias. The funnel plot of all polymorphisms was symmetrical. The funnel plot for the rs3754093 and rs1047840 polymorphisms in the allelic comparison was presented in Figs 5 and 6, respectively (rs3754093: G vs. A, Egger’s funnel plot, *P* > |t| = 0.349; rs1047840: G vs. A, Egger’s funnel plot, P > |t| = 0.337, Supplementary Table 4).

### LD analysis across populations

In order to better understand these results, LD analysis was performed to test for the existence of bins in the region comprising these eight polymorphisms (Meta-*P* value < 0.05/45). However, only six of them can be matched from the database, including rs1635498, rs1047840, rs9350, rs1635517, rs1776148 and rs1776177 polymorphisms. LD plots for TSI population presented high LD value (r^2^ = 0.70) between rs1635517 and rs1776177 polymorphisms, for CHB, JPT, CHD and GIH populations presented moderate LD values (r^2^ ≥ 0.50), while for YRI, ASW and LWK populations presented a lower LD value (r^2^ < 0.35) (Fig. 6 and Supplementary Table 5). LD plots for CHB, JPT, CHD, GIH, LWK and MEX populations presented a lower LD value (r^2^ < 0.35) between rs1776148 and rs9350 polymorphisms.

## Discussion

Multiple factors are involved in cancer formation and progression. Abundant evidence suggests that genetics play an important role in determining cancer susceptibility, and understanding associations between genetic polymorphisms and malignancies may provide personalized analysis and reveal the predictive value of certain carcinomas. An increasing number of research studies of tumourigenesis have highlighted EXO1 as a promising target.

The DNA repair system is known to be essential for maintaining genetic stability and offering protection from cancer initiation. EXO1 is an exclusive exonuclease gene that participates in the human MMR system. Genetic disorders in the MMR system result in the absence of DNA MMR function, resulting in an increased frequency of spontaneous mutations, which may give rise to the steady accumulation of oncogenes and tumour suppressors, which eventually contribute to tumourigenesis^20^.

For rs1047840, one polymorphism in EXO1 is located on exon 12, and its variation leads to a change of the 589^th^ amino acid of the Exo1 protein from lysine to glutamic acid, which might affect EXO1 expression. For the rs1047840 polymorphism, it is located in an exonic splicing enhancer (ESE) region^6^. Previous studies suggest that the A allele (variant allele) of the rs1047840 polymorphism may influence EXO1 activity, which would mildly affect its normal function^5^; in addition, as people who harbour the A allele (s) become older, transformations caused by carcinogens may accumulate through an increased number of unremoved DNA adducts. Thus, for an individual who has a risk-imparting genetic variant, such as the A allele (variant allele) of the rs1047840 polymorphism, that variant will likely synergistically increase their cancer risk.

Owing to the importance of EXO1 polymorphisms, an increasing number of studies have explored the associations of EXO1 polymorphisms and cancer risk. Luo *et al*.^20^ identified that the A allele of the EXO1 rs1047840 polymorphism was significantly related to an increased cervical cancer (CC) risk compared with the G allele (OR = 1.67, 95% CI: 1.13–2.45, *P* < 0.05). Similarly, many other molecular epidemiological studies are consistent with this association of the EXO1 rs1047840 with BC, LC, oral cancer (OC) and GC^9^,^10^,^23^,^25^. However, Zienolddiny *et al*.^7^ have shown no significant association of the rs1047840 polymorphism and non-small-cell gastric cancer (NSCGC) risk. Additionally, Song *et al*.^26^ indicated that the rs3902093 polymorphism was related to reduced expression of EXO1 (*P*_discovery_ = 6.6 × 10−4, *P*_replication_ = 0.039, *P*_joint_ = 2.5 × 10−4; OR_joint_ = 0.80, 95% CI: 0.71–0.90), while carriers of the A allele had lower expression (*P* = 0.002).

As these findings are not conclusive, we conducted the present comprehensive meta-analysis to shed light on the role of EXO1 polymorphisms in tumourigenesis. Unlike the previous work conducted by Duan *et al*.^27^, they only investigated one polymorphism in EXO1 and cancer risk and concluded that rs1047840 polymorphism A allele may be applied as a novel biomarker for tumour susceptibility. Recently, Chen *et al*.^28^ also conducted a meta-analysis encompassing three polymorphisms in EXO1 and cancer risk, and they concluded that rs9350 polymorphism was a protective factor against cancer, while the rs1047840 polymorphism may be a risk factor. Besides, the rs1776148 polymorphism may have no influence on cancer risk. In our present work, which considered 21,651 cancer patients and 21,348 cancer free controls, we concluded that the A allele of rs1047840 polymorphism conferred a significantly increased susceptibility to overall cancer in an allelic model, a result consistent with previous meta-analysis^27^,^28^. Similarly, the rs3754093, rs1776177, rs9350, rs10802996, rs1635498, rs1776148 and rs851797 polymorphisms were also identified related to an increased overall susceptibility to cancer in an allelic model, respectively, while no significant association was revealed for rs1635517 polymorphism. For rs9350 and rs1776148, our conclusions were not consistent with Chen *et al*.’s^27^ study, potentially because we excluded the studies that were deviated from HWE and our results were further adjusted by Bonferroni corrections^13^. In addition, when the stratification analyses were conducted by ethnicity and source of control, we identified a significantly increased susceptibility to Asian populations in all the genetic models of rs1047840 polymorphism, to Caucasians in an allelic model, and population-based (P-B) group in an allelic model. In addition, we uncovered an increased susceptibility to LC also in an allelic model. Moreover, we performed LD analyses to find the LD between the six polymorphisms, which showed that the rs1635517 and rs1776177 polymorphisms were in a high LD value (r^2^ = 0.70) for TSI population, and for CHB, JPT, CHD and GIH populations presented moderate LD values (r^2^ ≥ 0.50), while for YRI, ASW and LWK populations presented a lower LD value (r^2^ < 0.35). However, our meta-analysis results seemed not to be in accordance with the LD analyses that rs1776177 polymorphism is a risk factor for cancer rather than rs1635517. Limited number of studies and sample size may account for this discrepancy, therefore, future studies are warranted to verify this finding.

There are some strengths and limitations in the present study. The most important strength is that we have conducted a comprehensive retrieval for all eligible studies and polymorphisms, and the sample size was markedly expanded, which helped to reveal some findings not suggested in the previous work. Secondly, studies deviated from HWE were excluded, ensuring the accuracy of the finally data. Simultaneously, several limitation should be discussed. Firstly, the phenotype of our study is a heterogeneous aggregation of a variety of cancer types, and only for rs1047840 polymorphism, a subgroup analysis based on cancer type was conducted, while for others, attributing to the limited number of studies for specific cancers, such as BC, CC and *etc.*, we are unable to validate the potential effects on these cancers homogeneous or not. In addition, as ten case-control studies were excluded from the pooled analysis, there retained only two case-control studies for rs10802996 and rs1776148 polymorphisms, respectively. On the basis of small samples, possibly leading to an underestimate of the true association. All these confusions should be further confirmed in a series of much larger studies. Secondly, the effects of EXO1 polymorphisms on cancer susceptibility might be affected by several factors, such as age, sex, smoking status and matching criteria. A lack of samples and data may cause inconsistency in results and lead to possible publication bias, which often affect the precision of overall results. Thirdly, tumours are polygenic diseases, and analyses of haplotype and gene-to-gene associations are also important because gene-to-gene and gene-to-environment interactions may modulate cancer susceptibility.

In conclusion, our meta-analysis suggests that the rs1047840, rs9350, rs10802996, rs1635498, rs1776148, rs1776177, rs3754093 and rs851797 polymorphisms in EXO1 may be risk factors for cancer. In the future, large-scale well-designed case-control studies are warranted to verify our findings.

## Additional Information

**How to cite this article**: Zhang, M. *et al*. Associations between Nine Polymorphisms in EXO1 and Cancer Susceptibility: *A Systematic Review and Meta-Analysis of 39 Case-control Studies*. *Sci. Rep.* **6**, 29270; doi: 10.1038/srep29270 (2016).

## Supplementary Material

Supplementary Information

## Figures and Tables

**Table 1:** Characteristics of the enrolled studies on EXO1 polymorphisms and cancer.

SNP	First Author	Year	Ethnicity	Genotyping Method	Source of control	Cancer Type	Case			Control			
							AA	AB	BB	AA	AB	BB	Y/N (HWE)
rs9350	Haghighi *et al*.	2010	Asian	PCR-RFLP	HB	CRC	60	28	2	51	37	79	N
rs9350	Bau *et al*.	2009	Asian	PCR-RFLP	HB	GC	62	78	39	56	84	39	Y
rs9350	Wang *et al*.	2009	Asian	PCR-RFLP	HB	BC	433	563	276	402	596	274	Y
rs9350	Tsai *et al*.	2009	Asian	PCR-RFLP	HB	OC	235	297	148	214	313	153	Y
rs9350	Ibarrola-Villava *et al*.	2011	Caucasian	TaqMan	HB	Melanoma	485	186	13	297	99	10	Y
rs9350	Hsu *et al*.	2009	Asian	PCR-RFLP	HB	LC	124	156	78	112	167	79	Y
rs851797	Bau *et al*.	2009	Asian	PCR-RFLP	HB	GC	38	87	54	36	90	53	Y
rs851797	Wang *et al*.	2009	Asian	PCR-RFLP	HB	BC	266	625	381	258	630	384	Y
rs851797	Tsai *et al*.	2009	Asian	PCR-RFLP	HB	OC	139	328	213	133	344	203	Y
rs851797	Hsu *et al*.	2009	Asian	PCR-RFLP	HB	LC	76	173	109	72	179	107	Y
rs3754093	Bau *et al*.	2009	Asian	PCR-RFLP	HB	GC	68	83	28	75	82	22	Y
rs3754093	Wang *et al*.	2009	Asian	PCR-RFLP	HB	BC	504	599	169	535	577	160	Y
rs3754093	Tsai *et al*.	2009	Asian	PCR-RFLP	HB	OC	261	311	108	283	315	82	Y
rs3754093	Hsu *et al*.	2009	Asian	PCR-RFLP	HB	LC	135	167	56	149	164	45	Y
rs1776177	Bau *et al*.	2009	Asian	PCR-RFLP	HB	GC	80	82	17	82	84	13	Y
rs1776177	Wang *et al*.	2009	Asian	PCR-RFLP	HB	BC	581	587	104	589	592	91	N
rs1776177	Tsai *et al*.	2009	Asian	PCR-RFLP	HB	OC	309	306	65	319	308	53	Y
rs1776177	Hsu *et al*.	2009	Asian	PCR-RFLP	HB	LC	159	164	35	164	168	26	Y
rs1776148	Bau *et al*.	2009	Asian	PCR-RFLP	HB	GC	9	39	131	8	36	135	N
rs1776148	Wang *et al*.	2009	Asian	PCR-RFLP	HB	BC	64	267	941	59	255	958	N
rs1776148	Tsai *et al*.	2009	Asian	PCR-RFLP	HB	OC	35	148	497	31	138	511	N
rs1776148	Chang *et al*.	2008	Caucasian	SNP Chip	PB	Glioblastoma	11	57	44	18	47	46	Y
rs1776148	Ibarrola-Villava *et al*.	2011	Caucasian	PCR-RFLP	HB	Melanoma	67	293	239	48	171	160	Y
rs1776148	Hsu *et al*.	2009	Asian	PCR-RFLP	HB	LC	18	78	262	16	73	269	N
rs1635517	Bau *et al*.	2009	Asian	PCR-RFLP	HB	GC	11	65	103	8	59	112	Y
rs1635517	Wang *et al*.	2009	Asian	PCR-RFLP	HB	BC	52	449	771	54	421	797	Y
rs1635517	Tsai *et al*.	2009	Asian	PCR-RFLP	HB	OC	39	241	400	28	218	434	Y
rs1635517	Hsu *et al*.	2009	Asian	PCR-RFLP	HB	LC	22	130	206	15	118	225	Y
rs1635498	Bau *et al*.	2009	Asian	PCR-RFLP	HB	GC	132	43	4	137	39	3	Y
rs1635498	Wang *et al*.	2009	Asian	PCR-RFLP	HB	BC	968	289	15	972	283	17	Y
rs1635498	Tsai *et al*.	2009	Asian	PCR-RFLP	HB	OC	508	161	11	522	148	10	Y
rs1635498	Hsu *et al*.	2009	Asian	PCR-RFLP	HB	LC	265	85	8	274	79	5	Y
rs10802996	Luo *et al*.	2012	Asian	PCR-RFLP	HB	CC	77	39	10	172	89	17	Y
rs10802996	Bau *et al*.	2009	Asian	PCR-RFLP	HB	GC	100	59	20	102	61	16	Y
rs10802996	Wang *et al*.	2009	Asian	PCR-RFLP	HB	BC	735	408	129	728	426	118	N
rs10802996	Tsai *et al*.	2009	Asian	PCR-RFLP	HB	OC	380	235	65	383	235	62	N
rs10802996	Hsu *et al*.	2009	Asian	PCR-RFLP	HB	LC	199	119	40	203	122	33	N
rs1047840	Kabzińśki *et al*.	2014	Caucasian	TaqMan	PB	CRC	22	95	33	49	62	39	N
rs1047840	Nogueira *et al*.	2014	Mix	TaqMan	HB	HNSCC	179	209	62	175	211	64	Y
rs1047840	Tang *et al*.	2014	Mix	SNP Chip	HB	PC	827	910	296	815	993	304	Y
rs1047840	Luo *et al*.	2012	Asian	PCR-RFLP	HB	CC	73	48	5	196	77	5	Y
rs1047840	Tsai *et al*.	2009	Asian	PCR-RFLP	HB	OC	391	244	45	482	183	15	Y
rs1047840	Wang *et al*.	2009	Asian	PCR-RFLP	HB	BC	794	421	57	898	341	33	Y
rs1047840	Bau *et al*.	2009	Asian	PCR-RFLP	HB	GC	103	64	12	125	49	5	Y
rs1047840	Jin *et al*.	2008	Asian	SNP Chip	PB	LC	304	172	24	355	138	24	N
rs1047840	Zienolddiny *et al*.	2005	Caucasian	Beckman	HB	LC	115	106	35	116	145	30	Y
rs1047840	Hsu *et al*.	2009	Asian	PCR-RFLP	HB	LC	214	125	19	251	97	10	Y
rs1047840	Bayram *et al*.	2011	Caucasian	PCR-RFLP	HB	HCC	95	94	35	99	108	17	Y
rs1047840	Chang *et al*.	2008	Caucasian	SNP Chip	PB	Glioblastoma	55	42	15	29	59	22	Y
rs1047840	Ibarrola-Villava *et al*.	2011	Caucasian	PCR-RFLP	HB	Melanoma	234	282	83	136	175	68	Y

CRC: Colorectal cancer; GC: gastric cancer; BC: Breast cancer; OC: Oral cancer; LC: Lung cancer; CC: Cervical cancer; HNSCC: Head and eck squamous cell carcinoma; PC: Pancreatic cancer; HCC: Hepatic cellular cancer; H-B: Hospital based; P-B: Population based; HWE: Hardy Weinberg Equilibrium.

**Table 2:** Results of meta-analysis for polymorphisms in and cancer susceptibility.

Polymorphisms	Comparison	Subgroup	N	*P*_H_	*P*_Z_	Random	Fixed
rs1047840	B VS. A	Overall	11	0.000	**0.000**	4.082 (3.009–5.538)	3.571 (3.350–3.807)
	B VS. A	Asian	5	0.963	**0.000**	6.861 (6.127–7.682)	6.864 (6.130–7.687)
	B VS. A	Caucasian	4	0.028	**0.000**	2.709 (2.075–3.538)	2.654 (2.301–3.060)
	B VS. A	Mix	2	0.838	**0.000**	2.692 (2.452–2.955)	2.692 (2.452–2.955)
	B VS. A	PCR-RFLP	7	0.000	**0.000**	5.327 (3.631–7.815)	5.057 (4.600–5.560)
	B VS. A	SNP-Chip	2	0.175	**0.000**	2.488 (1.881–3.292)	2.662 (2.407–2.944)
	B VS. A	Other methods	2	0.434	**0.000**	2.778 (2.330–3.313)	2.778 (2.330–3.312)
	B VS. A	HB	10	0.000	**0.000**	4.357 (3.172–5.986)	3.615 (3.389–3.857)
	B VS. A	LC	2	0.000	**0.000**	4.510 (2.094–9.713)	4.382 (3.546–5.416)
	B VS. A	Other tumors	9	0.000	**0.000**	3.993 (2.824–5.647)	3.499 (3.272–3.742)
	BA VS. AA	Overall	11	0.000	0.396	1.091 (0.892–1.336)	1.100 (1.019–1.187)
	BA VS. AA	Asian	5	0.819	**0.000**	1.503 (1.335–1.691)	1.502 (1.335–1.691)
	BA VS. AA	Caucasian	4	0.048	0.071	0.748 (0.545–1.026)	0.799 (0.665–0.961)
	BA VS. AA	Mix	2	0.661	0.143	0.914 (0.811–1.031)	0.914 (0.811–1.031)
	BA VS. AA	PCR-RFLP	7	0.020	0.002	1.339 (1.117–1.605)	1.358 (1.223–1.508)
	BA VS. AA	SNP-Chip	2	0.005	0.261	0.612 (0.261–1.44)	0.866 (0.761–0.985)
	BA VS. AA	Other methods	2	0.243	0.229	0.865 (0.665–1.125)	0.873 (0.699–1.090)
	BA VS. AA	HB	10	0.000	0.112	1.165 (0.965–1.406)	1.121 (1.038–1.210)
	BA VS. AA	LC	2	0.004	0.870	1.061 (0.525–2.143)	1.098 (0.866–1.393)
	BA VS. AA	Other tumors	9	0.000	0.418	1.097 (0.877–1.372)	1.100 (1.015–1.192)
	BA + BB VS. AA	Overall	11	0.000	0.256	1.132 (0.914–1.401)	1.129 (1.051–1.214)
	BA + BB VS. AA	Asian	5	0.611	**0.000**	1.585 (1.414–1.776)	1.584 (1.414–1.775)
	BA + BB VS. AA	Caucasian	4	0.020	0.131	0.774 (0.555–1.080)	0.824 (0.693–0.980)
	BA + BB VS. AA	Mix	2	0.739	0.173	0.925 (0.826–1.035)	0.925 (0.826–1.035)
	BA + BB VS. AA	PCR-RFLP	7	0.002	**0.001**	1.405 (1.144–1.726)	1.413 (1.278–1.562)
	BA + BB VS. AA	SNP-Chip	2	0.002	0.270	0.609 (0.252–1.471)	0.877 (0.777–0.990)
	BA + BB VS. AA	Other methods	2	0.441	0.340	0.903 (0.732–1.114)	0.903 (0.732–1.114)
	BA + BB VS. AA	HB	10	0.000	0.052	1.219 (0.998–1.489)	1.152 (1.071–1.239)
	BA + BB VS. AA	LC	2	0.005	0.699	1.137 (0.593–2.179)	1.169 (0.931–1.467)
	BA + BB VS. AA	Other tumors	9	0.000	0.322	1.129 (0.888–1.437)	1.125 (1.042–1.214)
	BB VS. AA	Overall	11	0.000	0.068	1.379 (0.977–1.948)	1.139 (1.003–1.293)
	BB VS. AA	Asian	5	0.556	**0.000**	2.456 (1.818–3.317)	2.480 (1.839–3.344)
	BB VS. AA	Caucasian	4	0.002	0.778	0.914 (0.488–1.711)	0.903 (0.695–1.173)
	BB VS. AA	Mix	2	0.954	0.615	0.957 (0.808–1.135)	0.957 (0.808–1.135)
	BB VS. AA	PCR-RFLP	7	0.000	0.011	2.004 (1.176–3.414)	1.625 (1.309–2.018)
	BB VS. AA	SNP-Chip	2	0.019	0.350	0.636 (0.246–1.643)	0.910 (0.759–1.092)
	BB VS. AA	Other methods	2	0.535	0.894	1.022 (0.737–1.418)	1.022 (0.737–1.418)
	BB VS. AA	HB	10	0.000	0.014	1.529 (1.089–2.149)	1.175 (1.033–1.337)
	BB VS. AA	LC	2	0.193	0.097	1.519 (0.823–2.804)	1.462 (0.934–2.288)
	BB VS. AA	Other tumors	9	0.000	0.140	1.350 (0.907–2.011)	1.114 (0.976–1.272)
	BB VS. BA + AA	Overall	11	0.000	0.030	1.369 (1.031–1.819)	1.151 (1.022–1.298)
	BB VS. BA + AA	Asian	5	0.642	0.000	2.155 (1.600–2.904)	2.177 (1.618–2.929)
	BB VS. BA + AA	Caucasian	4	0.005	0.771	1.085 (0.626–1.881)	1.007 (0.789–1.284)
	BB VS. BA + AA	Mix	2	0.812	0.954	1.005 (0.858–1.176)	1.005 (0.858–1.176)
	BB VS. BA + AA	PCR-RFLP	7	0.000	0.015	1.832 (1.123–2.987)	1.489 (1.209–1.835)
	BB VS. BA + AA	SNP-Chip	2	0.189	0.867	0.900 (0.594–1.361)	0.986 (0.833–1.166)
	BB VS. BA + AA	Other methods	2	0.275	0.578	1.100 (0.784–1.542)	1.090 (0.804–1.478)
	BB VS. BA + AA	HB	10	0.000	0.011	1.460 (1.089–1.959)	1.173 (1.039–1.324)
	BB VS. BA + AA	LC	2	0.467	0.050	1.533 (0.995–2.362)	1.539 (1.000–2.367)
	BB VS. BA + AA	Other tumors	9	0.000	0.081	1.336 (0.965–1.852)	1.124 (0.992–1.273)
rs9350	B VS. A	Overall	5	0.000	0.000	2.930 (2.124–4.042)	2.656 (2.435–2.896)
	B VS. A	Asian	4	0.999	0.000	2.412 (2.201–2.643)	2.412 (2.201–2.643)
	B VS. A	PCR-RFLP	4	0.999	0.000	2.412 (2.201–2.643)	2.412 (2.201–2.643)
	BA VS. AA	Overall	5	0.513	0.105	0.908 (0.809–1.020)	0.909 (0.809–1.020)
	BA VS. AA	Asian	4	0.996	0.027	0.866 (0.762–0.983)	0.866 (0.762–0.983)
	BA VS. AA	PCR-RFLP	4	0.996	0.027	0.866 (0.762–0.983)	0.866 (0.762–0.983)
	BA + BB VS. AA	Overall	5	0.637	0.105	0.914 (0.820–1.019)	0.914 (0.820–1.019)
	BA + BB VS. AA	Asian	4	0.993	0.035	0.880 (0.782–0.991)	0.880 (0.782–0.991)
	BA + BB VS. AA	PCR-RFLP	4	0.993	0.035	0.880 (0.782–0.991)	0.880 (0.782–0.991)
	BB VS. AA	Overall	5	0.995	0.207	0.907 (0.780–1.055)	0.907 (0.780–1.055)
	BB VS. AA	Asian	4	0.989	0.236	0.911 (0.782–1.062)	0.911 (0.782–1.062)
	BB VS. AA	PCR-RFLP	4	0.989	0.236	0.911 (0.782–1.062)	0.911 (0.782–1.062)
	BB VS. BA + AA	Overall	5	0.978	0.816	0.984 (0.862–1.124)	0.984 (0.862–1.124)
	BB VS. BA + AA	Asian	4	0.991	0.891	0.991 (0.866–1.133)	0.991 (0.866–1.133)
	BB VS. BA + AA	PCR-RFLP	4	0.991	0.891	0.991 (0.866–1.133)	0.991 (0.866–1.133)

*P*_H_: *P* value of Q test for heterogeneity test; *P*_Z_: means statistically significant (P < 0.05); *P* (Adjust): Multiple testing *P* value according to Bonferroni Correction; LC: Lung cancer; H-B: Hospital based; P-B: Population based; HWE: Hardy Weinberg Equilibrium; *P* value less than 0.05/(9_polymorphisms_*5_models_) was considered as statistically significant, which was marked with bold font in the table). Note: Heterogeneity was considered to be significant when the *P*-value was less than 0.1. If there was no significant heterogeneity, a fixed effect model (Der-Simonian Laird) was used to evaluate the point estimates and 95% CI; otherwise, a random effects model (Der-Simonian Laird) was used. And the *P*z was calculated based on the actual model adopted.
