# Supplementary material for: Associations between Nine Polymorphisms in EXO1 and Cancer Susceptibility: A Systematic Review and Meta-Analysis of 39 Case-control Studies
Source: Sci Rep. 2016 Jul 8;6:29270. doi: 10.1038/srep29270 (PMC4937237; doi:10.1038/srep29270)
Supplement: Supplementary Information [file srep29270-s1.pdf]

Associations between Nine Polymorphisms in EXO1 and Cancer Susceptibility:

A Systematic Review and Meta-Analysis of 39 Case-control Studies

Meng Zhang[1,2], Duran Zhao[1,2], Cunye Yan[1,2], Li Zhang[1,2*], Chaozhao Liang[1,2*]

[1] Department of Urology, the First Affiliated Hospital of Anhui Medical University, Hefei, China

[2] Institute of Urology, Anhui Medical University, Hefei, China

Correspondence: Chaozhao Liang (liang_chaozhao@163.com); Li Zhang (lzhang_nanomed@163.com)

Department: Urology

Location: 218 Jixi Road, Shushan District, Hefei, China.

Tel.: +86-551-62923440

Fax: +86-551-63633742

Supplementary Information

Supplementary table 1. Methodological quality of the included studies according to the Newcastle-Ottawa Scale.

Supplementary table 2. Results of meta-analysis for polymorphisms in and cancer susceptibility.

Supplementary table 3. Details of the sensitivity analyses for the polymorphisms in EXO1 and cancer risk.

Supplementary table 4. *P* values of the Egger's test for the polymorphisms in EXO1.

Supplementary table 5. Details of the linkage disequilibrium analysis for EXO1 polymorphisms in populations from 1000 genomes Phase 3.

Supplementary figure 1. Begg's funnel plot for publication bias test under EXO1 rs1047840 polymorphism (allelic comparison G vs. A). The x-axis is log (OR), and the y-axis is natural logarithm of OR. The horizontal line in the figure represents the overall estimated log (OR). The two diagonal lines indicate the pseudo 95% confidence limits of the effect estimate. Log (OR) =log-transformed OR, OR=odds ratio.

Supplementary table 1. Methodological quality of the included studies according to the Newcastle-Ottawa Scale

**Variants**	**Author**	**Adequacy of Case Definition**	**Representativeness of the Cases**	**Selection of Controls**	**Definition of Controls**	**Comparability Cases/Controls**	**Ascertainment of Exposure**	**Same Method of Ascertainment**	**Non-response rate**
rs1047840	Kabziński *et al.*	*	*	NA	*	*	*	*	NA
	Nogueira *et al.*	*	*	NA	*	**	*	*	NA
	Tang *et al.*		*	*	*	*	**	*	NA
	Luo *et al.*	*	*	NA	*	*	*	.*	NA
	Tsai *et al.*	*	*	NA	*	*	*	*	NA
	Wang *et al.*	*	*	NA	*	**	*	*	NA
	Bau *et al.*	*	*	NA	*	**	*	*	NA
	Jin *et al.*	*	*	*	*	**	*	*	NA
	Zienolddiny *et al.*	*	*	*	*	**	*	*	NA
	Hsu *et al.*	*	*	NA	*	**	*	*	NA
	Bayram *et al.*	*	*	NA	*	*	*	*	NA
	Chang *et al.*	*	*	*	*	**	*	*	NA
	Ibarrola-Villava *et al.*	*	*	*	*	*	*	*	NA
rs1776148	Bau *et al.*	*	*	NA	*	**	*	*	NA
	Wang *et al.*	*	*	NA	*	**	*	*	NA
	Tsai *et al.*	*	*	NA	*	*	*	*	NA
	Chang *et al.*	*	*	*	*	**	*	*	NA
	Ibarrola-Villava *et al.*	*	*	*	*	*	*	*	NA
	Hsu *et al.*	*	*	NA	*	**	*	*	NA
rs9350	Haghighi *et al.*	NA	*	NA	*	NA	NA	*	NA

	Bau *et al.*	*	*	NA	*	**	*	*	NA
	Wang *et al.*	*	*	NA	*	**	*	*	NA
	Tsai *et al.*	*	*	NA	*	*	*	*	NA
	Ibarrola-Villava *et al.*	*	*	*	*	*	*	*	NA
	Hsu *et al.*	*	*	NA	*	**	*	*	NA
rs851797	Bau *et al.*	*	*	NA	*	**	*	*	NA
	Wang *et al.*	*	*	NA	*	**	*	*	NA
	Tsai *et al.*	*	*	NA	*	*	*	*	NA
	Hsu *et al.*	*	*	NA	*	**	*	*	NA
rs3754093	Bau *et al.*	*	*	NA	*	**	*	*	NA
	Wang *et al.*	*	*	NA	*	**	*	*	NA
	Tsai *et al.*	*	*	NA	*	*	*	*	NA
	Hsu *et al.*	*	*	NA	*	**	*	*	NA
rs1776177	Bau *et al.*	*	*	NA	*	**	*	*	NA
	Wang *et al.*	*	*	NA	*	**	*	*	NA
	Tsai *et al.*	*	*	NA	*	*	*	*	NA
	Hsu *et al.*	*	*	NA	*	**	*	*	NA
rs10802996	Luo *et al.*	*	*	NA	*	*	*	.*	NA
	Bau *et al.*	*	*	NA	*	**	*	*	NA
	Wang *et al.*	*	*	NA	*	**	*	*	NA
	Tsai *et al.*	*	*	NA	*	*	*	*	NA
	Hsu *et al.*	*	*	NA	*	**	*	*	NA
rs1635517	Bau *et al.*	*	*	NA	*	**	*	*	NA
	Wang *et al.*	*	*	NA	*	**	*	*	NA

	Tsai *et al.*	*	*	NA	*	*	*	*	NA
	Hsu *et al.*	*	*	NA	*	**	*	*	NA
rs1635498	Bau *et al.*	*	*	NA	*	**	*	*	NA
	Wang *et al.*	*	*	NA	*	**	*	*	NA
	Tsai *et al.*	*	*	NA	*	*	*	*	NA
	Hsu *et al.*	*	*	NA	*	**	*	*	NA

This table identifies ‘high’ quality choices with a ‘star’. A study can be awarded a maximum of 1 star for each numbered item within the Selection and Exposure categories. A maximum of 2 stars can be given for Comparability. *, Yes; NA, not applicable. (http://www.ohri.ca/programs/clinical_epidemiology/oxford.htm).

Supplementary table 2. Results of meta-analysis for polymorphisms in and cancer susceptibility.

Polymorphisms	Comparison	Subgroup	N	P_H	P_Z	Random	Fixed
rs1635498	B VS. A	Overall	4	0.983	**0.000**	7.965 (6.924-9.163)	7.967 (6.926-9.164)
	BB VS. AA	Overall	4	0.811	0.671	1.103 (0.692-1.759)	1.106 (0.696-1.758)
	BA VS. AA	Overall	4	0.934	0.313	1.071 (0.937-1.223)	1.071 (0.937-1.223)
	BA+BB VS.AA	Overall	4	0.879	0.289	1.073 (0.942-1.222)	1.073 (0.942-1.222)
	BB VS.BA+AA	Overall	4	0.827	0.724	1.085 (0.681-1.728)	1.087 (0.684-1.726)
rs1635517	B VS. A	Overall	4	0.939	0.019	1.128 (1.020-1.247)	1.128 (1.020-1.247)
	BB VS. AA	Overall	4	0.480	0.102	0.800 (0.610-1.048)	0.799 (0.610-1.045)
	BA VS. AA	Overall	4	0.677	0.564	0.923 (0.699-1.218)	0.922 (0.699-1.216)
	BA+BB VS.AA	Overall	4	0.534	0.199	0.841 (0.644-1.099)	0.840 (0.644-1.096)
	BB VS.BA+AA	Overall	4	0.752	0.010	0.862 (0.769-0.966)	0.862 (0.769-0.966)
rs3754093	B VS. A	Overall	4	0.895	**0.000**	2.976 (2.711-3.268)	2.976 (2.711-3.268)
	BB VS. AA	Overall	4	0.651	0.011	1.256 (1.054-1.497)	1.257 (1.055-1.497)
	BA VS. AA	Overall	4	0.995	0.128	1.097 (0.974-1.237)	1.097 (0.974-1.237)
	BA+BB VS.AA	Overall	4	0.979	0.033	1.132 (1.010-1.267)	1.132 (1.010-1.267)
	BB VS.BA+AA	Overall	4	0.574	0.031	1.196 (1.016-1.409)	1.197 (1.017-1.409)
rs851797	B VS. A	Overall	4	0.995	**0.000**	1.841 (1.686-2.009)	1.841 (1.686-2.009)
	BB VS. AA	Overall	4	0.997	0.746	0.974 (0.831-1.142)	0.974 (0.831-1.142)
	BA VS. AA	Overall	4	0.989	0.394	0.938 (0.811-1.086)	0.938 (0.811-1.086)
	BA+BB VS.AA	Overall	4	0.999	0.482	0.952 (0.829-1.092)	0.952 (0.829-1.092)
	BB VS.BA+AA	Overall	4	0.959	0.758	1.019 (0.903-1.150)	1.019 (0.903-1.150)
rs10802996	B VS. A	Overall	2	0.726	**0.000**	5.013 (3.717-6.762)	5.003 (3.708-6.751)
	BB VS. AA	Overall	2	0.957	0.354	1.291 (0.753-2.216)	1.291 (0.752-2.216)
	BA VS. AA	Overall	2	0.981	0.916	0.983 (0.711-1.358)	0.983 (0.711-1.358)

	BA+BB VS.AA	Overall	2	0.965	0.799	1.040 (0.770-1.404)	1.040 (0.770-1.404)
	BB VS.BA+AA	Overall	2	0.953	0.331	1.299 (0.767-2.200)	1.299 (0.766-2.200)
rs1776148	B VS. A	Overall	2	0.797	**0.000**	1.448 (1.209-1.734)	1.448 (1.209-1.734)
	BB VS. AA	Overall	2	0.435	0.456	1.152 (0.790-1.682)	1.154 (0.792-1.682)
	BA VS. AA	Overall	2	0.317	0.111	1.349 (0.928-1.960)	1.352 (0.933-1.959)
	BA+BB VS.AA	Overall	2	0.341	0.204	1.254 (0.879-1.787)	1.257 (0.883-1.788)
	BB VS.BA+AA	Overall	2	0.984	0.430	0.910 (0.719-1.151)	0.910 (0.719-1.151)
rs1776177	B VS. A	Overall	3	0.994	**0.000**	3.234 (2.815-3.716)	3.234 (2.815-3.716)
	BB VS. AA	Overall	3	0.963	0.074	1.311 (0.974-1.765)	1.311 (0.974-1.765)
	BA VS. AA	Overall	3	0.993	0.848	1.016 (0.861-1.200)	1.016 (0.861-1.200)
	BA+BB VS.AA	Overall	3	0.998	0.489	1.058 (0.902-1.241)	1.058 (0.902-1.241)
	BB VS.BA+AA	Overall	3	0.951	0.071	1.301 (0.977-1.730)	1.301 (0.978-1.730)

P_H: *P* value of Q test for heterogeneity test; P_Z: means statistically significant (P＜0.05); ***P* (Adjust)**: Multiple testing *P* value according to Bonferroni Correction; **LC**: Lung cancer; **H-B**: Hospital based; **P-B**: Population based; **HWE**: Hardy Weinberg Equilibrium; The bold print of the *P* values represents statistically significant (Before adjustment and after adjustment); ***P*** value less than 0.05/ ($9_{polymorphisms}$*5_{models}) was considered as statistically significant, which was marked with bold font in the table). **Note:** Heterogeneity was considered to be significant when the *P*-value was less than 0.1. If there was no significant heterogeneity, a fixed effect model (Der-Simonian Laird) was used to evaluate the point estimates and 95%CI; otherwise, a random effects model (Der-Simonian Laird) was used. And the *P*z was calculated based on the actual model adopted.

Supplementary table 3. Details of the sensitivity analyses for the polymorphisms in EXO1 and cancer risk.

SNP	Comparison	Study omitted	Estimate	[95% Confident Interval]	Effect Model
rs9350	T VS. C	Bau et al. (2009)	3.060	2.095-4.470	Random
		Wang et al. (2009)	3.094	1.937-4.942	
		Tsai et al. (2009)	3.100	1.987-4.838	
		Ibarrola-Villava et al. (2011)	2.412	2.201-2.643	
		Hsu et al. (2009)	3.084	2.058-4.620	
		Combined	2.930	2.124-4.042	
	TT VS. CC	Bau et al. (2009)	0.908	0.776-1.061	Fixed
		Wang et al. (2009)	0.881	0.713-1.089	
		Tsai et al. (2009)	0.917	0.769-1.094	
		Ibarrola-Villava et al. (2011)	0.911	0.782-1.062	
		Hsu et al. (2009)	0.910	0.773-1.071	
		Combined	0.907	0.780-1.055	
	TC VS. CC	Bau et al. (2009)	0.913	0.810-1.029	Fixed
		Wang et al. (2009)	0.933	0.800-1.087	
		Tsai et al. (2009)	0.922	0.808-1.052	
		Ibarrola-Villava et al. (2011)	0.866	0.762-0.983	
		Hsu et al. (2009)	0.918	0.811-1.038	
		Combined	0.909	0.809-1.020	
	TT VS. TC+CC	Bau et al. (2009)	0.983	0.857-1.128	Fixed
		Wang et al. (2009)	0.960	0.797-1.158	
		Tsai et al. (2009)	0.994	0.851-1.161	
		Ibarrola-Villava et al. (2011)	0.991	0.866-1.133	
		Hsu et al. (2009)	0.984	0.853-1.136	
		Combined	0.984	0.862-1.124	
	TT+TC VS. CC	Bau et al. (2009)	0.918	0.821-1.027	Fixed
		Wang et al. (2009)	0.929	0.804-1.072	
		Tsai et al. (2009)	0.928	0.820-1.050	
		Ibarrola-Villava et al. (2011)	0.880	0.782-0.991	
		Hsu et al. (2009)	0.922	0.821-1.035	
		Combined	0.914	0.820-1.019	
rs851797	T VS. C	Bau et al. (2009)	1.840	1.680-2.015	Fixed
		Wang et al. (2009)	1.864	1.644-2.113	
		Tsai et al. (2009)	1.830	1.651-2.028	
		Hsu et al. (2009)	1.838	1.672-2.021	
		Combined	1.841	1.686-2.009	
	TT VS. CC	Bau et al. (2009)	0.975	0.826-1.150	Fixed
		Wang et al. (2009)	0.987	0.785-1.239	
		Tsai et al. (2009)	0.963	0.799-1.160	
		Hsu et al. (2009)	0.976	0.821-1.159	
		Combined	0.974	0.831-1.142	
	TC VS. CC	Bau et al. (2009)	0.940	0.808-1.094	Fixed

		Wang et al. (2009)	0.914	0.741-1.127	
		Tsai et al. (2009)	0.948	0.799-1.125	
		Hsu et al. (2009)	0.942	0.805-1.104	
		Combined	0.938	0.811-1.086	
	TT VS. TC+CC	Bau et al. (2009)	1.019	0.898-1.155	Fixed
		Wang et al. (2009)	1.052	0.885-1.250	
		Tsai et al. (2009)	1.000	0.868-1.153	
		Hsu et al. (2009)	1.018	0.893-1.160	
		Combined	1.019	0.903-1.150	
	TT+TC VS. CC	Bau et al. (2009)	0.953	0.826-1.100	Fixed
		Wang et al. (2009)	0.941	0.772-1.146	
		Tsai et al. (2009)	0.954	0.812-1.121	
		Hsu et al. (2009)	0.955	0.823-1.108	
		Combined	0.952	0.829-1.092	
rs3754093	G VS. A	Bau et al. (2009)	2.968	2.694-3.271	Fixed
		Wang et al. (2009)	3.089	2.703-3.530	
		Tsai et al. (2009)	2.925	2.622-3.264	
		Hsu et al. (2009)	2.966	2.681-3.281	
		Combined	2.976	2.711-3.268	
	GG VS. AA	Bau et al. (2009)	1.246	1.038-1.494	Fixed
		Wang et al. (2009)	1.408	1.099-1.805	
		Tsai et al. (2009)	1.196	0.973-1.470	
		Hsu et al. (2009)	1.237	1.023-1.496	
		Combined	1.257	1.055-1.497	
	GA VS. AA	Bau et al. (2009)	1.096	0.968-1.241	Fixed
		Wang et al. (2009)	1.093	0.920-1.298	
		Tsai et al. (2009)	1.108	0.963-1.274	
		Hsu et al. (2009)	1.093	0.960-1.244	
		Combined	1.097	0.974-1.237	
	GG+GA VS. AA	Bau et al. (2009)	1.128	1.003-1.269	Fixed
		Wang et al. (2009)	1.159	0.985-1.363	
		Tsai et al. (2009)	1.127	0.987-1.287	
		Hsu et al. (2009)	1.124	0.995-1.270	
		Combined	1.132	1.010-1.267	
	GG VS. GA+AA	Bau et al. (2009))	1.160	0.979-1.374	Fixed
		Wang et al. (2009))	1.289	1.024-1.622	
		Tsai et al. (2009))	1.114	0.919-1.350	
		Hsu et al. (2009))	1.155	0.968-1.378	
		Combined	1.168	0.992-1.375	
rs1776177	G VS. A	Bau et al. (2009)	3.242	2.789-3.769	Fixed
		Tsai et al. (2009)	3.208	2.604-3.951	
		Hsu et al. (2009)	3.242	2.747-3.825	
		Combined	3.234	2.815-3.716	
	GG VS. AA	Bau et al. (2009)	1.306	0.947-1.801	Fixed

		Tsai et al. (2009)	1.372	0.874-2.156	
		Hsu et al. (2009)	1.281	0.900-1.823	
		Combined	1.311	0.974-1.765	
	GA VS. AA	Bau et al. (2009)	1.019	0.851-1.220	Fixed
		Tsai et al. (2009)	1.005	0.782-1.290	
		Hsu et al. (2009)	1.020	0.837-1.244	
		Combined	1.016	0.861-1.200	
	GG+GA VS. AA	Bau et al. (2009)	1.060	0.892-1.260	Fixed
		Tsai et al. (2009)	1.054	0.829-1.340	
		Hsu et al. (2009)	1.058	0.875-1.279	
		Combined	1.058	0.902-1.241	
	GG VS. GA+AA	Bau et al. (2009)	1.266	0.930-1.723	Fixed
		Tsai et al. (2009)	1.333	0.864-2.057	
		Hsu et al. (2009)	1.242	0.885-1.743	
		Combined	1.272	0.956-1.692	
rs1776148	G VS. A	Chang et al. (2008)	1.431	1.171-1.749	Fixed
		Ibarrola-Villava et al. (2011)	1.519	1.008-2.288	
		Combined	1.448	1.209-1.734	
	GG VS. AA	Chang et al. (2008)	1.070	0.702-1.631	Fixed
		Ibarrola-Villava et al. (2011)	1.565	0.665-3.686	
		Combined	1.154	0.792-1.682	
	GA VS. AA	Chang et al. (2008)	1.228	0.810-1.861	Fixed
		Ibarrola-Villava et al. (2011)	1.985	0.854-4.614	
		Combined	1.352	0.933-1.959	
	GG+GA VS. AA	Chang et al. (2008)	1.151	0.776-1.710	Fixed
		Ibarrola-Villava et al. (2011)	1.777	0.798-3.960	
		Combined	1.257	0.883-1.788	
	GG VS. GA+AA	Chang et al. (2008)	1.494	1.136-1.965	Fixed
		Ibarrola-Villava et al. (2011)	0.957	0.559-1.637	
		Combined	1.362	1.067-1.738	
rs1635517	T VS. C	Bau et al. (2009)	1.130	1.018-1.254	Fixed
		Wang et al. (2009)	1.091	0.945-1.259	
		Tsai et al. (2009)	1.142	1.016-1.285	
		Hsu et al. (2009)	1.135	1.018-1.265	
		Combined	1.128	1.020-1.247	
	TT VS. CC	Bau et al. (2009)	0.811	0.612-1.074	Fixed
		Wang et al. (2009)	0.651	0.449-0.946	
		Tsai et al. (2009)	0.862	0.626-1.186	
		Hsu et al. (2009)	0.836	0.624-1.122	
		Combined	0.799	0.610-1.045	
	TC VS. CC	Bau et al. (2009)	0.933	0.699-1.246	Fixed
		Wang et al. (2009)	0.782	0.533-1.148	
		Tsai et al. (2009)	0.979	0.705-1.359	
		Hsu et al. (2009)	0.958	0.708-1.295	

		Combined	0.922	0.699-1.216	
	TT+TC VS. CC	Bau et al. (2009)	0.852	0.646-1.125	Fixed
		Wang et al. (2009)	0.696	0.481-1.005	
		Tsai et al. (2009)	0.902	0.658-1.236	
		Hsu et al. (2009)	0.877	0.656-1.172	
		Combined	0.840	0.644-1.096	
	TT VS. TC+CC	Bau et al. (2009)	0.946	0.839-1.067	Fixed
		Wang et al. (2009)	0.920	0.779-1.086	
		Tsai et al. (2009)	0.952	0.832-1.090	
		Hsu et al. (2009)	0.949	0.837-1.075	
		Combined	0.944	0.841-1.060	
rs1635498	G VS. A	Bau et al. (2009)	7.944	6.868-9.189	Fixed
		Wang et al. (2009)	8.182	6.711-9.976	
		Tsai et al. (2009)	7.929	6.726-9.348	
		Hsu et al. (2009)	7.898	6.786-9.192	
		Combined	7.967	6.926-9.164	
	GG VS. AA	Bau et al. (2009)	1.080	0.664-1.758	Fixed
		Wang et al. (2009)	1.317	0.706-2.458	
		Tsai et al. (2009)	1.096	0.633-1.898	
		Hsu et al. (2009)	1.016	0.609-1.693	
		Combined	1.106	0.696-1.758	
	GA VS. AA	Bau et al. (2009)	1.065	0.928-1.223	Fixed
		Wang et al. (2009)	1.120	0.926-1.354	
		Tsai et al. (2009)	1.054	0.901-1.232	
		Hsu et al. (2009)	1.064	0.921-1.229	
		Combined	1.071	0.937-1.223	
	GG+GA VS. AA	Bau et al. (2009)	1.066	0.931-1.220	Fixed
		Wang et al. (2009)	1.133	0.941-1.363	
		Tsai et al. (2009)	1.056	0.907-1.230	
		Hsu et al. (2009)	1.061	0.922-1.221	
		Combined	1.073	0.942-1.222	
	GG VS. GA+AA	Bau et al. (2009)	1.062	0.653-1.727	Fixed
		Wang et al. (2009)	1.277	0.686-2.380	
		Tsai et al. (2009)	1.079	0.624-1.867	
		Hsu et al. (2009)	1.000	0.600-1.664	
		Combined	1.085	0.683-1.724	
rs10802996	G VS. C	Luo et al. (2012)	4.781	3.206-7.130	Fixed
		Bau et al. (2009)	5.326	3.391-8.363	
		Combined	5.003	3.708-6.751	
	GG VS. CC	Luo et al. (2012)	1.275	0.625-2.601	Fixed
		Bau et al. (2009)	1.314	0.575-3.001	
		Combined	1.291	0.752-2.216	
	GC VS. CC	Luo et al. (2012)	0.987	0.628-1.550	Fixed
		Bau et al. (2009)	0.979	0.616-1.554	

		Combined	0.983	0.711-1.358	
	GG+GC VS. CC	Luo et al. (2012)	1.046	0.689-1.589	Fixed
		Bau et al. (2009)	1.033	0.670-1.591	
		Combined	1.040	0.770-1.404	
	GG VS. GC+CC	Luo et al. (2012)	1.250	0.626-2.498	Fixed
		Bau et al. (2009)	0.588	0.264-1.309	
		Combined	0.900	0.538-1.507	
rs1047840	G VS. A	Nogueira et al. (2014)	4.274	3.062-5.965	Random
		Tang et al. (2014)	4.270	3.056-5.967	
		Luo et al. (2012)	3.914	2.847-5.381	
		Tsai et al. (2009)	3.833	2.837-5.179	
		Wang et al. (2009)	3.867	2.877-5.198	
		Bau et al. (2009)	3.888	2.832-5.337	
		Zienolddiny et al. (2005)	4.204	3.021-5.850	
		Hsu et al. (2009)	3.886	2.830-5.336	
		Bayram et al. (2011)	4.116	2.960-5.723	
		Chang et al. (2008)	4.357	3.172-5.986	
		Ibarrola-Villava et al. (2011)	4.340	3.137-6.004	
		Combined	4.082	3.009-5.538	
	GG VS. AA	Nogueira et al. (2014)	1.456	0.984-2.154	Random
		Tang et al. (2014)	1.471	0.961-2.250	
		Luo et al. (2012)	1.334	0.937-1.900	
		Tsai et al. (2009)	1.225	0.894-1.679	
		Wang et al. (2009)	1.322	0.918-1.905	
		Bau et al. (2009)	1.317	0.926-1.872	
		Zienolddiny et al. (2005)	1.411	0.965-2.063	
		Hsu et al. (2009)	1.324	0.925-1.896	
		Bayram et al. (2011)	1.320	0.920-1.893	
		Chang et al. (2008)	1.529	1.089-2.149	
		Ibarrola-Villava et al. (2011)	1.504	1.034-2.188	
		Combined	1.379	0.977-1.948	
	GA VS. AA	Nogueira et al. (2014)	1.104	0.884-1.378	Random
		Tang et al. (2014)	1.117	0.895-1.394	
		Luo et al. (2012)	1.054	0.855-1.299	
		Tsai et al. (2009)	1.042	0.851-1.277	
		Wang et al. (2009)	1.057	0.848-1.318	
		Bau et al. (2009)	1.059	0.858-1.307	
		Zienolddiny et al. (2005)	1.134	0.919-1.398	
		Hsu et al. (2009)	1.055	0.853-1.306	
		Bayram et al. (2011)	1.109	0.894-1.376	
		Chang et al. (2008)	1.165	0.965-1.406	
		Ibarrola-Villava et al. (2011)	1.108	0.888-1.382	
		Combined	1.091	0.892-1.336	
	GG+GA VS. AA	Nogueira et al. (2014)	1.150	0.910-1.453	Random

	Tang et al. (2014)	1.160	0.917-1.467	
	Luo et al. (2012)	1.092	0.875-1.363	
	Tsai et al. (2009)	1.076	0.872-1.327	
	Wang et al. (2009)	1.098	0.868-1.389	
	Bau et al. (2009)	1.093	0.875-1.365	
	Zienolddiny et al. (2005)	1.169	0.933-1.465	
	Hsu et al. (2009)	1.094	0.873-1.371	
	Bayram et al. (2011)	1.136	0.903-1.430	
	Chang et al. (2008)	1.219	0.998-1.489	
	Ibarrola-Villava et al. (2011)	1.163	0.924-1.464	
	Combined	1.132	0.914-1.401	
GG VS. GA+AA	Nogueira et al. (2014)	1.422	1.077-1.877	Random
	Tang et al. (2014)	1.436	1.098-1.877	
	Luo et al. (2012)	1.366	1.058-1.762	
	Tsai et al. (2009)	1.224	0.993-1.509	
	Wang et al. (2009)	1.306	1.008-1.692	
	Bau et al. (2009)	1.315	1.026-1.686	
	Zienolddiny et al. (2005)	1.378	1.050-1.808	
	Hsu et al. (2009)	1.318	1.022-1.700	
	Bayram et al. (2011)	1.294	1.008-1.660	
	Chang et al. (2008)	1.418	1.101-1.827	
	Ibarrola-Villava et al. (2011)	1.381	1.039-1.836	
	Combined	1.349	1.055-1.724	

Supplementary table 4. *P* values of the Egger's test for the polymorphisms in EXO1.

**Polymorphism**	**Subgroup**	**Egger's test** $\boldsymbol{P > |t|}$
rs1047840	Overall	0.337
	Asian	0.957
	Caucasian	0.816
	PCR-RFLP	0.566
	H-B	0.371
rs9350	Overall	0.430
	Asian	0.572
	PCR-RFLP	0.722
rs851797	Overall	0.406
rs3754093	Overall	0.349
rs1776177	Overall	0.142
rs1635517	Overall	0.310
rs1635498	Overall	0.145

PCR-RFLP: polymerase chain reaction-restriction fragment length polymorphism; H-B: hospital-based.

Supplementary table 5. Details of the linkage disequilibrium analysis for EXO1 polymorphisms in populations from 1000 genomes Phase 3.

L1	L2	D'	LOD	r^2	CI-low	CI-hi	Distance
**ASW**							
rs1635517	rs1776177	1.000	5.390	0.316	0.690	1.000	116
rs1635517	rs1047840	0.378	0.470	0.048	0.050	0.700	30268
rs1635517	rs1776148	0.326	0.800	0.064	0.070	0.580	30512
rs1635517	rs1635498	0.256	0.340	0.031	0.030	0.590	33242
rs1635517	rs9350	0.687	0.670	0.042	0.110	0.910	36641
rs1776177	rs1047840	0.119	0.190	0.014	0.000	0.340	30152
rs1776177	rs1776148	0.251	0.400	0.035	0.030	0.530	30396
rs1776177	rs1635498	0.416	0.270	0.029	0.040	0.790	33126
rs1776177	rs9350	0.511	0.890	0.078	0.110	0.790	36525
rs1047840	rs1776148	0.085	0.050	0.004	0.000	0.400	244
rs1047840	rs1635498	0.587	0.840	0.058	0.120	0.850	2974
rs1047840	rs9350	0.210	0.080	0.008	0.020	0.680	6373
rs1776148	rs1635498	0.311	0.310	0.030	0.030	0.660	2730
rs1776148	rs9350	0.722	0.900	0.086	0.150	0.920	6129
rs1635498	rs9350	1.000	0.930	0.051	0.140	0.990	3399
**CEU**							
rs1776177	rs1047840	0.537	5.240	0.170	0.350	0.680	30152
rs1776177	rs1776148	0.633	6.450	0.211	0.440	0.770	30396
rs1776177	rs1635498	1.000	1.200	0.022	0.200	1.000	33126
rs1776177	rs9350	0.046	0.010	0.000	0.000	0.400	36525
rs1047840	rs1776148	0.128	0.410	0.015	0.010	0.290	244
rs1047840	rs1635498	0.599	0.510	0.013	0.080	0.890	2974
rs1047840	rs9350	0.506	2.440	0.091	0.240	0.700	6373
rs1776148	rs1635498	0.266	0.030	0.001	0.030	0.850	2730
rs1776148	rs9350	0.397	0.560	0.017	0.060	0.690	6129
rs1635498	rs9350	0.651	0.030	0.002	0.040	0.960	3399
**CHD**							
rs1635517	rs1776177	1.000	14.940	0.634	0.870	1.000	116
rs1635517	rs1047840	0.248	0.960	0.059	0.060	0.440	30268
rs1635517	rs1776148	0.014	0.000	0.000	-0.010	0.250	30512
rs1635517	rs1635498	0.669	3.920	0.246	0.400	0.840	33242
rs1635517	rs9350	0.698	1.470	0.070	0.230	0.890	36641
rs1776177	rs1047840	0.286	0.970	0.054	0.070	0.490	30152
rs1776177	rs1776148	0.291	1.140	0.061	0.080	0.490	30396
rs1776177	rs1635498	0.604	2.140	0.127	0.270	0.810	33126
rs1776177	rs9350	0.600	1.570	0.082	0.220	0.810	36525
rs1047840	rs1776148	0.212	0.740	0.041	0.040	0.400	244
rs1047840	rs1635498	0.383	1.050	0.077	0.100	0.630	2974

rs1047840	rs9350	0.533	0.800	0.041	0.110	0.800	6373
rs1776148	rs1635498	1.000	0.450	0.021	0.080	0.980	2730
rs1776148	rs9350	1.000	4.420	0.165	0.660	1.000	6129
rs1635498	rs9350	1.000	2.080	0.074	0.380	1.000	3399
**GIH**							
rs1635517	rs1776177	1.000	16.850	0.531	0.890	1.000	116
rs1635517	rs1047840	0.391	1.630	0.093	0.150	0.580	30268
rs1635517	rs1776148	0.298	1.960	0.086	0.130	0.440	30512
rs1635517	rs1635498	0.226	0.120	0.006	0.020	0.650	33242
rs1635517	rs9350	0.190	0.130	0.007	0.010	0.550	36641
rs1776177	rs1047840	0.632	3.030	0.132	0.350	0.800	30152
rs1776177	rs1776148	0.544	4.160	0.167	0.340	0.700	30396
rs1776177	rs1635498	0.035	0.000	0.000	0.000	0.500	33126
rs1776177	rs9350	0.078	0.060	0.002	0.000	0.350	36525
rs1047840	rs1776148	0.177	0.470	0.018	0.020	0.370	244
rs1047840	rs1635498	0.236	0.040	0.002	0.020	0.830	2974
rs1047840	rs9350	0.110	0.030	0.001	0.010	0.540	6373
rs1776148	rs1635498	1.000	1.660	0.069	0.290	1.000	2730
rs1776148	rs9350	0.914	5.230	0.177	0.630	0.980	6129
rs1635498	rs9350	1.000	0.900	0.023	0.140	0.990	3399
**CHB**							
rs1635517	rs1776177	1.000	14.980	0.570	0.870	1.000	116
rs1635517	rs1047840	0.273	1.130	0.062	0.070	0.460	30268
rs1635517	rs1776148	0.170	0.020	0.002	0.020	0.810	30512
rs1635517	rs1635498	0.511	4.010	0.214	0.310	0.670	33242
rs1635517	rs9350	0.599	1.680	0.067	0.230	0.810	36641
rs1776177	rs1047840	0.229	0.710	0.037	0.040	0.430	30152
rs1776177	rs1776148	0.445	2.230	0.104	0.200	0.640	30396
rs1776177	rs1635498	0.445	1.870	0.096	0.180	0.650	33126
rs1776177	rs9350	0.653	3.910	0.133	0.400	0.810	36525
rs1047840	rs1776148	0.195	0.400	0.028	0.020	0.420	244
rs1047840	rs1635498	0.407	1.960	0.113	0.170	0.600	2974
rs1047840	rs9350	0.326	0.590	0.024	0.050	0.600	6373
rs1776148	rs1635498	1.000	0.840	0.043	0.130	0.990	2730
rs1776148	rs9350	0.898	4.270	0.135	0.580	0.970	6129
rs1635498	rs9350	1.000	5.150	0.154	0.690	1.000	3399
**JPT**							
rs1635517	rs1776177	1.000	15.520	0.557	0.880	1.000	116
rs1635517	rs1047840	0.112	0.260	0.011	0.000	0.300	30268
rs1635517	rs1776148	0.216	0.750	0.038	0.040	0.400	30512
rs1635517	rs1635498	0.669	3.580	0.227	0.400	0.830	33242
rs1635517	rs9350	0.803	2.850	0.156	0.440	0.930	36641
rs1776177	rs1047840	0.408	1.920	0.083	0.170	0.600	30152
rs1776177	rs1776148	0.569	3.290	0.149	0.320	0.740	30396

rs1776177	rs1635498	0.633	2.070	0.112	0.280	0.830	33126
rs1776177	rs9350	0.579	2.870	0.145	0.310	0.750	36525
rs1047840	rs1776148	0.539	5.720	0.263	0.360	0.680	244
rs1047840	rs1635498	0.020	0.000	0.000	0.000	0.310	2974
rs1047840	rs9350	0.707	2.310	0.109	0.340	0.880	6373
rs1776148	rs1635498	1.000	1.410	0.048	0.240	1.000	2730
rs1776148	rs9350	1.000	6.860	0.204	0.760	1.000	6129
rs1635498	rs9350	1.000	1.430	0.122	0.240	1.000	3399
**LWK**							
rs1635517	rs1776177	1.000	8.560	0.283	0.810	1.000	116
rs1635517	rs1047840	0.014	0.000	0.000	0.000	0.360	30268
rs1635517	rs1776148	0.294	1.790	0.074	0.120	0.450	30512
rs1635517	rs1635498	0.331	0.390	0.021	0.040	0.660	33242
rs1635517	rs9350	0.293	0.110	0.008	0.030	0.760	36641
rs1776177	rs1047840	0.056	0.030	0.001	0.000	0.350	30152
rs1776177	rs1776148	0.521	1.510	0.066	0.190	0.740	30396
rs1776177	rs1635498	1.000	0.590	0.055	0.090	0.980	33126
rs1776177	rs9350	0.369	0.860	0.042	0.080	0.620	36525
rs1047840	rs1776148	0.094	0.160	0.007	0.000	0.290	244
rs1047840	rs1635498	0.386	0.650	0.028	0.070	0.680	2974
rs1047840	rs9350	0.174	0.220	0.010	0.010	0.460	6373
rs1776148	rs1635498	0.359	0.730	0.029	0.070	0.640	2730
rs1776148	rs9350	1.000	2.810	0.076	0.500	1.000	6129
rs1635498	rs9350	0.478	0.050	0.004	0.040	0.960	3399
**MEX**							
rs1776177	rs1047840	0.099	0.040	0.003	0.000	0.470	30152
rs1776177	rs1776148	0.886	3.970	0.218	0.550	0.970	30396
rs1776177	rs1635498	1.000	0.500	0.017	0.080	0.980	33126
rs1776177	rs9350	0.197	0.240	0.013	0.020	0.490	36525
rs1047840	rs1776148	0.126	0.180	0.013	0.010	0.370	244
rs1047840	rs1635498	1.000	1.090	0.053	0.170	0.990	2974
rs1047840	rs9350	0.170	0.090	0.005	0.010	0.560	6373
rs1776148	rs1635498	1.000	0.270	0.008	0.050	0.980	2730
rs1776148	rs9350	1.000	3.640	0.154	0.590	1.000	6129
rs1635498	rs9350	1.000	0.320	0.010	0.060	0.980	3399
**TSI**							
rs1635517	rs1776177	1.000	23.250	0.707	0.920	1.000	116
rs1635517	rs1047840	0.149	0.270	0.013	0.010	0.370	30268
rs1635517	rs1776148	0.489	3.640	0.176	0.290	0.640	30512
rs1635517	rs1635498	0.140	0.020	0.001	0.020	0.730	33242
rs1635517	rs9350	0.037	0.000	0.000	0.000	0.550	36641
rs1776177	rs1047840	0.351	1.050	0.052	0.090	0.570	30152
rs1776177	rs1776148	0.791	7.730	0.324	0.600	0.900	30396
rs1776177	rs1635498	1.000	1.140	0.027	0.180	0.990	33126

rs1776177	rs9350	0.083	0.020	0.001	0.010	0.530	36525
rs1047840	rs1776148	0.278	1.280	0.063	0.080	0.450	244
rs1047840	rs1635498	0.631	0.600	0.025	0.100	0.900	2974
rs1047840	rs9350	0.250	0.350	0.018	0.030	0.550	6373
rs1776148	rs1635498	0.642	0.650	0.021	0.100	0.900	2730
rs1776148	rs9350	1.000	2.360	0.077	0.430	1.000	6129
rs1635498	rs9350	1.000	0.280	0.004	0.060	0.980	3399
**YRI**							
rs1635517	rs1776177	1.000	18.490	0.326	0.890	1.000	116
rs1635517	rs1047840	0.155	0.280	0.007	0.010	0.380	30268
rs1635517	rs1776148	0.429	5.290	0.144	0.280	0.560	30512
rs1635517	rs1635498	0.713	8.160	0.223	0.510	0.850	33242
rs1635517	rs9350	0.755	3.250	0.060	0.430	0.900	36641
rs1776177	rs1047840	0.274	2.240	0.054	0.120	0.410	30152
rs1776177	rs1776148	0.567	5.760	0.137	0.380	0.710	30396
rs1776177	rs1635498	0.762	3.840	0.081	0.460	0.900	33126
rs1776177	rs9350	0.747	8.160	0.180	0.550	0.870	36525
rs1047840	rs1776148	0.033	0.030	0.001	-0.010	0.220	244
rs1047840	rs1635498	0.913	5.370	0.112	0.630	0.980	2974
rs1047840	rs9350	0.786	6.400	0.143	0.560	0.900	6373
rs1776148	rs1635498	0.426	2.390	0.061	0.200	0.620	2730
rs1776148	rs9350	1.000	8.220	0.137	0.800	1.000	6129
rs1635498	rs9350	1.000	3.180	0.045	0.560	1.000	3399

Population descriptors: ASW: African ancestry in Southwest USA, CEU: Utah residents with Northern and Western European ancestry from the CEPH collection, CHB: Han Chinese in Beijing, China, CHD: Chinese in Metropolitan Denver, Colorado, GIH: Gujarati Indians in Houston, Texas, JPT: Japanese in Tokyo, Japan, LWK: Luhya in Webuye, Kenya, MEX: Mexican ancestry in Los Angeles, California, MKK: Maasai in Kinyawa, Kenya, TSI: Toscans in Italy, YRI: Yoruba in Ibadan, Nigeria. The linkage disequilibrium values were calculated using $r2$ and D' statistic; CI (Confidence Interval); LOD: Log odds score.

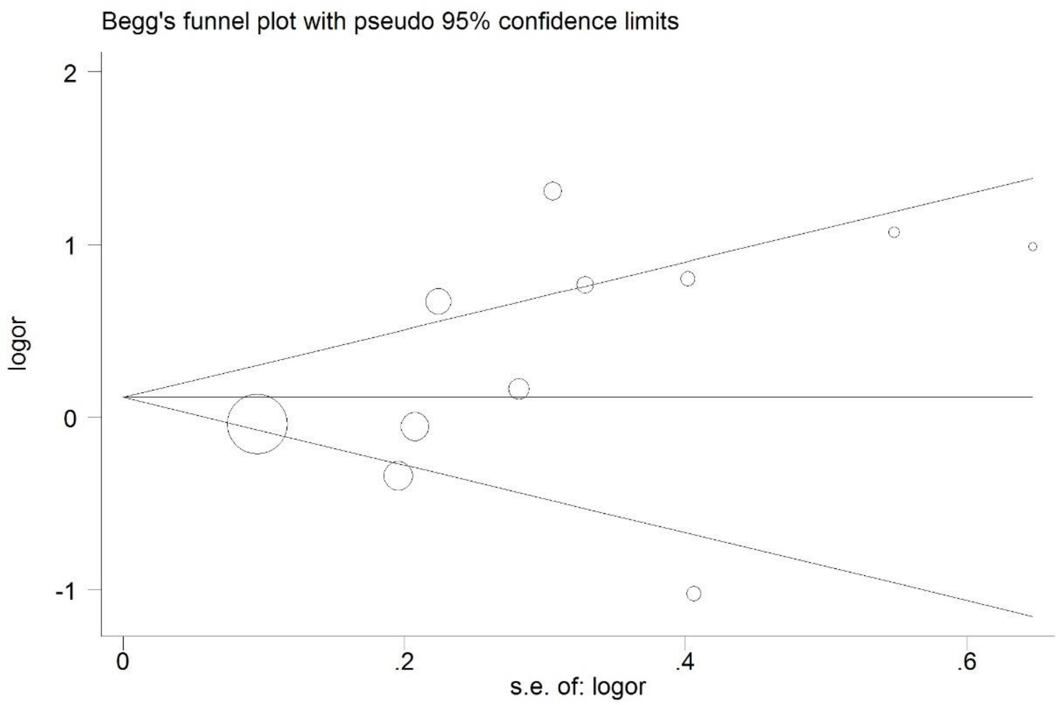

Supplementary figure 1. Begg’s funnel plot for publication bias test under EXO1 rs1047840 polymorphism (allelic comparison G vs. A). The x-axis is log (OR), and the y-axis is natural logarithm of OR. The horizontal line in the figure represents the overall estimated log (OR). The two diagonal lines indicate the pseudo 95% confidence limits of the effect estimate. Log (OR) =log-transformed OR, OR=odds ratio.
